# Requirement for integrin-linked kinase in neural crest migration and differentiation and outflow tract morphogenesis

**DOI:** 10.1186/1741-7007-11-107

**Published:** 2013-10-16

**Authors:** Xiuqin Dai, Weijian Jiang, Qingquan Zhang, Lian Xu, Peng Geng, Shaowei Zhuang, Brian G Petrich, Cizhong Jiang, Luying Peng, Shoumo Bhattacharya, Sylvia M Evans, Yunfu Sun, Ju Chen, Xingqun Liang

**Affiliations:** 1 Key Laboratory of Arrhythmia, Ministry of Education, East Hospital, Tongji University School of Medicine, 150 Jimo Road, Shanghai 200120, China; 2 New Era Stroke Care and Research Center, The Second Artillery General Hospital PLA, Beijing 100088, China; 3 Department of Medicine, University of California San Diego, 9500 Gilman Drive, La Jolla, CA 92093, USA; 4 School of Life Sciences and Technology, Tongji University, Shanghai 200120, China; 5 Department of Cardiovascular Medicine, University of Oxford, Oxford, UK

**Keywords:** ILK, Cardiac neural crest, Outflow tract, Migration, Congenital heart disease

## Abstract

**Background:**

Neural crest defects lead to congenital heart disease involving outflow tract malformation. Integrin-linked-kinase (ILK) plays important roles in multiple cellular processes and embryogenesis. ILK is expressed in the neural crest, but its role in neural crest and outflow tract morphogenesis remains unknown.

**Results:**

We ablated ILK specifically in the neural crest using the Wnt1-Cre transgene. ILK ablation resulted in abnormal migration and overpopulation of neural crest cells in the pharyngeal arches and outflow tract and a significant reduction in the expression of neural cell adhesion molecule (NCAM) and extracellular matrix components. ILK mutant embryos exhibited an enlarged common arterial trunk and ventricular septal defect. Reduced smooth muscle differentiation, but increased ossification and neurogenesis/innervation were observed in ILK mutant outflow tract that may partly be due to reduced transforming growth factor β2 (TGFβ2) but increased bone morphogenetic protein (BMP) signaling. Consistent with these observations, microarray analysis of fluorescence-activated cell sorting (FACS)-sorted neural crest cells revealed reduced expression of genes associated with muscle differentiation, but increased expression of genes of neurogenesis and osteogenesis.

**Conclusions:**

Our results demonstrate that ILK plays essential roles in neural crest and outflow tract development by mediating complex crosstalk between cell matrix and multiple signaling pathways. Changes in these pathways may collectively result in the unique neural crest and outflow tract phenotypes observed in ILK mutants.

## Background

Cardiac outflow tract (OFT) formation involves complex signaling and cell interactions, in which the neural crest plays a central role [1,2]. Neural crest cells (NCCs) delaminate from the dorsal neural tube and migrate along distinct pathways to their final destinations, where they differentiate into a plethora of cell types including melanocytes, cranial cartilage/bone, vascular smooth muscle cells (SMCs) and peripheral neurons. Cardiac NCCs, a subpopulation of NCCs from the dorsal neural tube between the mid-otic placode and the third somite, migrate and populate in pharyngeal arches 3, 4 and 6 and contribute to patterning of the pharyngeal arch arteries. A subset of cardiac NCCs continue migrating into the heart and are required for OFT septation [1]. Perturbation of cardiac NCC development causes congenital heart defects, including DiGeorge syndrome. However, the molecular mechanisms underlying NCC and OFT development are incompletely understood [1,3].

Multiple signaling pathways are implicated in cardiac NCC development, including bone morphogenetic protein (BMP), transforming growth factor β2 (TGFβ2), Notch, Wnt and platelet-derived growth factor (PDGF). Mutation of individual genes in these pathways variably affects NCC migration, proliferation, SMC differentiation and survival, leading to a spectrum of OFT abnormalities [1,2]. TGFβ2 signaling is required for SMC differentiation and extracellular matrix (ECM) deposition. Ablation of TGFβR1 or TGFβR2 leads to increased cell death within the OFT [4-7]. Mice deficient for BMP receptor ALK2 exhibit impaired NCC migration into the proximal OFT and SMC differentiation [8]. Deletion of Smad4, a common downstream mediator of TGFβ and BMP signaling, in cardiac NCCs leads to increased cell death in the pharyngeal arches and reduced NCCs within the OFT [9]. Double deletion of PDGF receptors leads to reduced migration of cardiac NCCs into the proximal OFT and persistent truncus arteriosus (PTA) [10]. However, mutation of each of the aforementioned genes has minimal effects on cardiac NCC delamination and initial migration into the pharyngeal arches and OFT.

NCC migration involves changes in cell-cell and cell-matrix interactions and cytoskeleton organization that determine the timing, velocity and pattern of NCC migration. Integrins are cell surface receptors required for cell adhesion, migration, proliferation and differentiation [11]. Integrins are recruited into focal adhesion complexes where they signal through interactions with focal adhesion proteins including focal adhesion kinase (FAK), integrin-linked kinase (ILK), PINCH ('Particularly Interesting New Cys-His protein’), parvin and paxillin [12]. Interactions of focal adhesion proteins with integrins are important for focal adhesion formation and maturation, modulation of integrin-ligand binding affinity and ECM remodeling [11,12]. Deletion of focal adhesion protein PINCH1 or FAK in NCCs results in defective TGFβ signaling and impaired SMC differentiation of mutant cardiac NCCs. However, migration of FAK or PINCH1 mutant cardiac NCCs into the OFT is normal [13,14]. Interestingly, PINCH1 mutant embryos presented a hyperplastic common arterial trunk that is distinct from that seen in FAK mutants, suggesting distinct roles in NCC and OFT morphogenesis.

ILK, a key component of focal adhesion complex, binds to the cytoplasmic domain of β1 and β3 integrins and several key actin binding proteins and adaptors [12]. ILK has been shown to act as a serine/threonine kinase to directly phosphorylate and activate downstream signaling molecules including Akt and glycogen synthase kinase 3β (GSK3β) [12,15,16]. However, a recent *in vivo* study has shown that the kinase activity of ILK is dispensable; instead, the pseudokinase domain of ILK acts as a protein interaction domain essential for recruitment of several adaptors and signaling molecules, including parvins [17,18]. Genetic studies have revealed an essential role for ILK during embryogenesis [19-21]. Deletion of ILK in mice leads to peri-implantation lethality, which resembles that of integrin β1 or PINCH1 knockouts [21-24]. Genetic studies in different contexts have revealed distinct requirements for ILK in ECM deposition/assembly, migration, proliferation and survival [25-29]. Due to early embryonic lethality of ILK null mice, the role of ILK in NCC and OFT development is unknown.

In this study, we ablated ILK specifically in NCC using the Wnt1-Cre transgene. Deletion of ILK in NCC resulted in abnormal NCC migration to the pharyngeal arches and the OFT and impaired smooth muscle differentiation. ILK mutant embryos present an enlarged common arterial trunk similar to that of PINCH1 mutants, but strikingly different from that of other mutants reported previously, suggesting a unique role of ILK-PINCH1 in cardiac NCC and a previously unappreciated role for cardiac NCC in OFT formation.

## Results

### Generation of mice with ILK deletion specifically in the neural crest cells

We analyzed expression of ILK in the OFT during development. RNA *in situ* hybridization (ISH) with a probe to ILK revealed ubiquitous expression of ILK in mouse embryos at E8.5 and E9.5 (Additional file 1: Figure S1A,B,B’). Immunostaining with ILK antibody and β-galactosidase (β-gal) staining of adjacent sections from Wnt1-Cre; Rosa-LacZ embryos at E10.5 showed that ILK was expressed in the OFT, including the OFT mesenchyme that was colocalized with Wnt1-Cre lineage (β-gal+) (Additional file 1: Figure S1C-E). Furthermore, immunostaining of cultured NCCs with antibodies to ILK and Sox10, a neural crest marker, revealed expression of ILK in the cytoplasm and focal adhesions of NCCs (Additional file 1: Figure S1F-H).

To investigate the role of ILK in NCC and OFT morphogenesis, we ablated ILK specifically in NCCs using Wnt1-Cre. Floxed ILK (ILK^f/f^) [27] mice were crossed with *Wnt1-Cre* mice. Resulting ILK heterozygous mice (Wnt1-Cre;ILK^f/+^) were viable, fertile and did not present any phenotypes, and thus were used as a littermate control in this study. Wnt1-Cre;ILK^f/+^ mice were backcrossed with ILK^f/f^ mice to generate NCC-specific ILK mutant mice (NKO). Ablation of ILK expression in neural crest derivatives of the OFT was confirmed by ILK immunostaining (Additional file 1: Figure S1I-L).

### Deletion of ILK in NCCs resulted in embryonic lethality and OFT malformation

**Figure 1:**
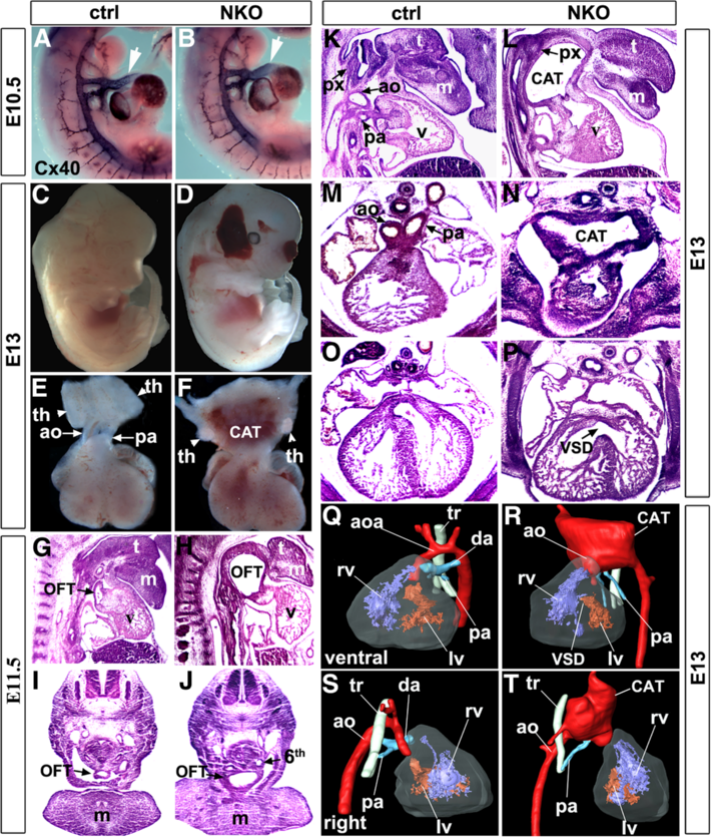
**Outflow tract (OFT) malformation in neural crest cell (NCC)-specific integrin-linked kinase (ILK) knockout (NKO) mutant mice. (A,B)***In situ* hybridization with a probe to connexin 40 (Cx40) showing an enlarged OFT in E10.5 NKO mutant embryo (arrow). **(C,D)** Cranial hemorrhage in the nasal and temporal regions of NKO mutant head at E13. **(E,F)** An enlarged common arterial trunk (CAT) and hypoplastic thymus (th, arrowhead) in NKO mutants at E13, compared to a well-defined aorta (ao) and pulmonary artery (pa) in control littermates. **(G-J)** A markedly enlarged OFT in E11.5 NKO mutant embryos that protrudes in between the tongue and pharynx ((G,H), saggital section; (I,J), transection; 6th, sixth arch artery). **(K-P)** An enlarged CAT, ventricular septal defect (VSD), thinner ventricular myocardium, hypoplastic mandible (m) and deformed pharynx (px) in NKO mutant embryos at E13 ((K,L), saggital section; (M-P), transection). **(Q-T)** MRI and 3D reconstruction of control and NKO mutant hearts at E13 ((Q,R), ventral view; (S,T) right view) show VSD, right ventricular outflow tract (RVOT) and a large CAT in NKO mutants. *aoa* aortic arch, *da* ductus arteriosus, *lv* left ventricle, *rv* right ventricle, *t* tongue, *tr* trachea.

To visualize the morphology of the OFT and pharyngeal arch arteries, we did ISH analysis of NKO and control littermates at E10.5 with a probe to connexin 40 (C×40) that is expressed in vascular endothelial cells, thus outlining the blood vessels [30]. At E10.5, the pharyngeal arteries of NKO mutant embryos displayed a pattern similar to that of control littermates (Figure 1A,B). However, a notable dilation of NKO mutant OFT was observed at this early stage (Figure 1B, arrow). From E11.5 onwards, NKO mutant embryos exhibited progressive OFT dilation, hypoplastic thymus and frequent cranial hemorrhage (Figure 1C-F). All NKO mutant embryos died around E13.5. At E13, control littermates exhibited well-defined aorta and pulmonary arteries, but NKO mutant OFT embryos displayed an enlarged common trunk that was stiff and difficult to separate from surrounding tissues (Figure 1E,F, common arterial trunk (CAT)). Histological analysis of control (Figure 1G,I,K,M,O) and NKO mutant (Figure 1H,J,L,N,P) embryos at E11.5 and E13 revealed that NKO mutant OFTs were markedly dilated and protruded in between the tongue and pharynx, pushing the tongue ventrally, and the pharynx of NKO mutants became deformed at E13 (Figure 1L, arrow). Furthermore, we observed ventricular septal defect (VSD), a thinner ventricular myocardium compact zone (Figure 1O,P) and shortened mandibles (Figure 1K,L) in NKO mutants at E13. Furthermore, MRI and 3D reconstruction of control and NKO mutant hearts at E13 revealed severe cardiovascular defects in NKO mutant embryos, including VSD and right ventricular outflow tract (RVOT) and a markedly dilated CAT that appeared to be formed by inclusion of all segments of the OFT and proximal parts of the pharyngeal arch arteries (Figure 1Q-T).

### Premature emigration and altered migration pattern of cardiac NCC in NKO mutant embryos

**Figure 2:**
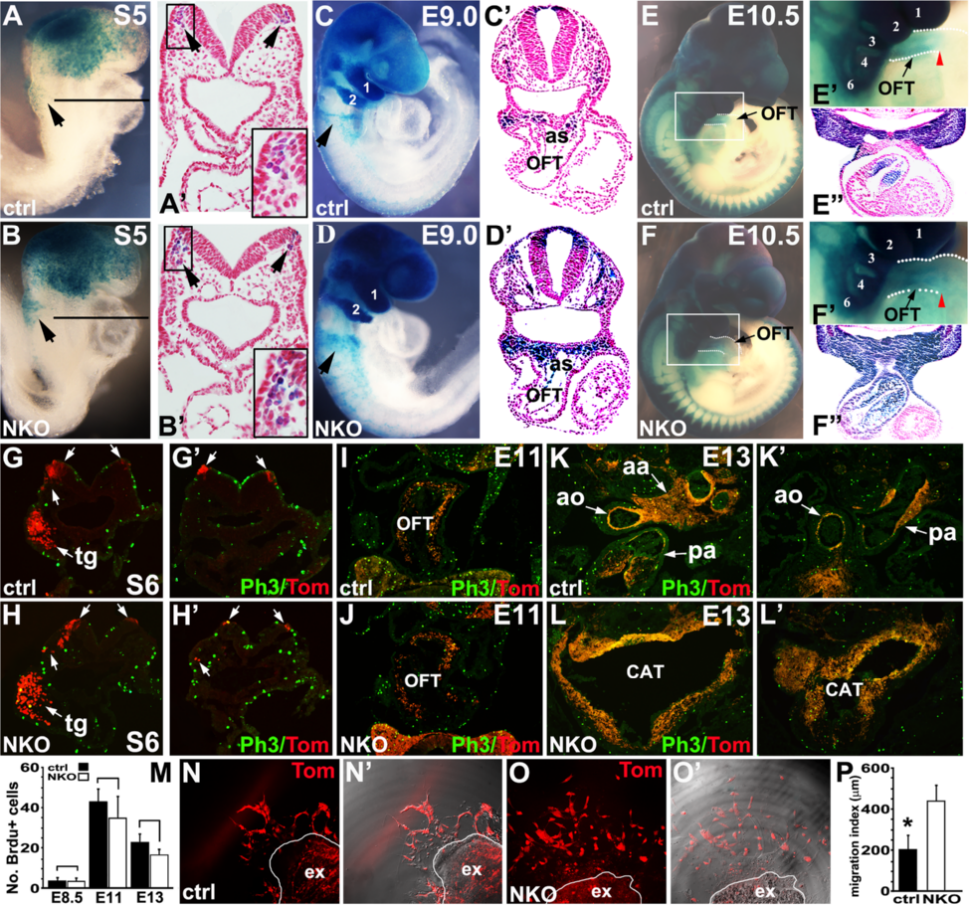
**Fate mapping analyses of neural crest cell (NCC) migration in control and NCC-specific integrin-linked kinase (ILK) knockout (NKO) embryos. (A-B’)** β-Galactosidase (β-gal) staining at E8.5 (5 somites) shows a slightly increased number of NCCs in the first pharyngeal arch and premature migration of postotic NCCs ((B,B’), arrow, inset). **(C-D’)** The caudal stream of postotic NCCs in NKO mutant mice seems wider compared to control embryos at E9.0 ((C,D), arrow) and there is significant accumulation of NCCs in the cranial region, the pharyngeal arches and circumpharyngeal region (C,D), some of which have prematurely migrated into aortic sac (as) and the outflow tract (OFT) (C’,D’). **(E,F”)** Compared to control littermates at E10.5 **(E’,E”)**, there are significantly increased NKO mutant NCCs in the pharyngeal arches 3 to 6. NKO mutant NCCs migrate beyond the conotruncal junction and populate entire layers of the OFT ((F’,F”) red arrowhead). **(G-M)** immunostaining with phosphohistone H3 (Ph3) antibody revealed a similar rate of proliferation in NCCs (tdTomato+) in the dorsal neural tube and surrounding mesenchyme at E8.5 (S6) (G-H’), and in NCC derived cells (tdTomato+) within the OFTs at E11 (I,J) and E13 (K-L’). Note that, in NKO mutant embryos at E8.5 (S6) (H,H’), there were a significantly increased number of cranial NCCs migrating into paraxial mesenchyme (H), and premature postotic NCC migration (H’). It should also be noted that the sections shown in (G) and (H) are tilted. NKO mutant hearts at E13 exhibit enlarged CAT compared to control littermates ((K,L), aortic arch/distal OFT; (K’,L’) proximal OFT) (*aa* aortic arch, *ao* aorta, *pa* pulmonary artery, *tg* trigeminal neural crest). Quantitative analysis of Ph3+/tdTomato + cells (mitotic index) (M). **(N-O’)** Neural tube explants (ex) cultures show abnormal migration of NKO mutant NCCs (O,O’) compared to control NCCs. Panels (N’) and (O’), phase contrast and tdTomato Red merging pictures. **(P)** Quantitative analysis of migration index/areas of NKO mutant and control NCCs.

Wnt1-Cre lineage analysis was performed to examine the migration and colonization of NKO mutant cardiac NCCs into the pharyngeal arches and OFT. Wnt1-Cre; ILK^f/+^ mice were crossed with ILK^f/f^ mice on a Rosa-LacZ [31] or Rosa-tdTomato [32] reporter background that allows us to visualize NCCs by β-gal staining and tdTomato Red. At E8.5 (5 to 6 somites, S5, S6), a slightly increased number of β-gal+/tdTomato + cells were observed in the cranial mesenchyme and the forming first pharyngeal arch (trigeminal neural crest-tg) of NKO mutants (Figure 2A,B,G,H, tg). Few, if any, control postotic NCCs were found in the paraxial mesoderm underneath the neural plates (Figure 2A-A’, arrow, inset; 2G’). In contrast, a substantial number of postotic NCCs of somite-matched NKO mutant embryos at E8.5 (S5) were found to migrate to the paraxial mesenchyme (Figure 2B-B’, arrow, inset; 2H’), suggesting ablation of ILK resulted in premature NCC emigration. Control cardiac NCCs at E9 (16 to 18 somites) migrated as distinct streams en route to pharyngeal arch 3, 4, 6 and the OFT, a few of which migrated into the aortic sac (Figure 2C,C’, arrow). In contrast, a significant increase in the number of NKO mutant NCCs at E9 (somite-matched) was observed in cranial regions and the first and second pharyngeal arches (Figure 2D). Migration of postotic NCCs of NKO mutants appeared to be diffuse and the caudal stream seemed slightly wider in the NKO mice (Figure 2D, arrow). Histological analysis revealed an increased number of NCCs accumulated in the pharyngeal arches and aortic sac in NKO mutant embryos at E9.0 and some NCCs had prematurely migrated into the OFT (Figure 2D’). At E10.5, cardiac NCCs of control embryos started to migrate into the OFT up to the conotruncal junction and colonized the subendocardial mesenchyme of the outflow cushion (Figure 2E,E’,E”, red arrowhead). In somite-matched NKO mutants, the OFT and the third pharyngeal arch were significantly enlarged and a marked increase in the number of NCCs was found in the pharyngeal mesoderm. Within the OFT, NKO mutant NCCs migrated beyond the conotruncal junction and invaded entire layers of the OFT (Figure 2F,F’,F”, red arrowhead). At E13, histological analysis of the aortic arch and proximal OFT revealed that the OFT of control littermates were separated into aorta and pulmonary arteries, and control NCCs contributed to a subpopulation of mesenchymal cells (tdTomato Red+) of aortic arch, aorta, pulmonary artery and OFT cushion (Figure 2K,K’). However, the OFT of NKO mutants at E13 exhibited an enlarged common trunk with a significantly increased number of NCCs (tdTomato Red+) (Figure 2L,L’).

An increased number of NCCs in the pharyngeal arches and OFT of NKO mutant embryos may be due to increased NCC proliferation and reduced cell death. We examined NCC proliferation by phosphohistone H3 (Ph3) antibody staining at E8.5 (Figure 2G,H, cranial level; G’,H’, postotic level), E11 (Figure 2I,J) and E13 (Figure 2K-L’). Mitotic indices were determined by counting the number of cells per section that were doubly positive for tdTomato Red (NCCs) and Ph3 staining, normalized to the total number of NCCs and derivatives (tdTomato+) within the pharyngeal arteries and OFTs of NKO mutant and control embryos (Figure 2M). We did not observe significant change in proliferation in the pharyngeal and OFT regions of NKO and control littermates. We also performed terminal deoxynucleotidyl transferase dUTP nick-end labeling (TUNEL) staining to measure neural crest cell death and no significant change in cell death in the pharyngeal and OFT regions of NKO and control littermates was observed at different developmental stages (data not shown).

We further examined the rate and pattern of NCC migration in neural crest explant culture from E8.5 NKO mutant and control embryos. NKO mutant NCCs on fibronectin coated plates were able to migrate out of neural tube explants. However, after 24 h, most NKO mutant NCCs rounded up and detached, suggesting defective adhesion. We cultured neural crest explants embedded in mitrogel. After 24 h, some control NCCs (tdTomato Red+) started to migrate out as distinct streams (Figure 2N,N’). However, NKO mutant NCCs migrated earlier (12 to 24 h after incubation) and lost their stream-like migration pattern as individual cells (Figure 2O,O’). Quantitative analysis of NCC migration [33] revealed that NKO mutant NCCs migrated significantly longer distances and to a bigger outgrowth area (Figure 2P) compared to control NCCs.

### Reduced focal adhesion and Akt phosphorylation, and impaired cytoskeleton organization in NKO mutant NCCs

**Figure 3:**
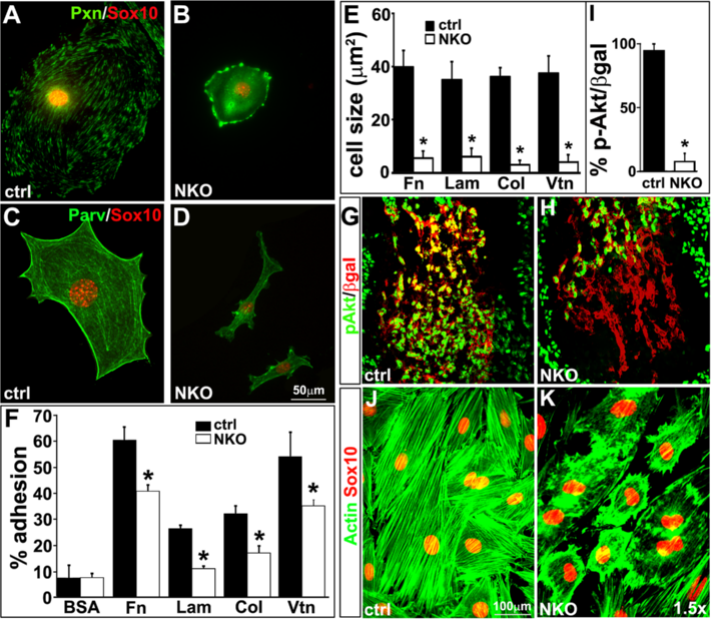
**Reduced cell adhesion and disorganized cytoskeleton of neural crest cell (NCC)-specific integrin-linked kinase (ILK) knockout (NKO) mutant NCCs. (A-D)** In control NCCs, paxillin and parvin are expressed in a punctate pattern, associated with the focal adhesions. However, in ILK mutant NCCs, paxillin expression is diffuse, while parvin expression appears to be normal. **(E,F)** Adhesion and spreading of ILK mutant NCCs (ILK^f/f^;Cre^+^) on fibronectin (Fn), laminin (Lam), collagen (Col) and vitronectin (Vtn) are impaired. **(G-I)** Reduced Akt phosphorylation in NCCs of NKO mutant OFT. **(J,K)** Immunostaining with antibodies to actin and Sox10 show well-aligned actin filaments in control NCCs. However, ILK^f/f^;Cre^+^ mutant NCCs exhibit excessive small protrusions and a large accumulation of cortical actin in the cell cortex.

To examine the formation of focal adhesion complexes of mutant NCCs in culture, we examined expression of paxillin and parvin, two key components of the focal adhesion complex. In control NCCs, expression of paxillin and parvin displayed a well organized pattern associated with focal adhesions (Figure 3A,C). In NKO mutant NCCs, expression of paxillin in focal adhesions appeared to be diffuse. However, expression of parvin and PINCH1 were relatively normal revealed by immunostaining and western blot (Figure 3B,D, and data not shown).

NCCs express multiple integrin subtypes with differential binding affinity to distinct ECM ligands. To test whether deletion of ILK selectively affected the adhesive function mediated by particular integrins, we assayed adhesion of NKO mutant and control NCCs to different integrin ligands. Because of difficulty of isolating enough NCCs from NKO mutant embryos, we established an ILK^f/f^ NCC culture and ablated ILK expression *in vitro* by infection of Cre recombinase adenoviruses [28]. For this experiment, ILK^f/f^ NCCs were infected with Cre or control adenoviruses, and cultured for additional 48 h and then used to examine adhesion and spreading of ILK mutant (ILK^f/f^;Cre^+^) and control NCCs (ILK^f/f^;Cre^-^) on different substrates. ILK mutant NCCs on all substrates tested exhibited progressive cell shrinkage (Figure 3E,K) and detachment during the course of culture. Adhesions of ILK mutant NCCs to fibronectin, laminin, collagen and vitronectin were markedly reduced (Figure 3F) suggesting that in NCCs, ILK is required for adhesion to multiple classes of integrins.

Activation of integrins leads to Akt phosphorylation and cytoskeleton reorganization required for cell growth, survival and migration. Coimmunostaining with antibodies to β-gal and phosphorylated Akt revealed a significant reduction in Akt phosphorylation in NKO mutant NCCs compared to the control (Figure 3G-I). In culture, control NCCs were polarized with well-aligned actin filaments. However, in ILK mutant NCCs 48-h post-viral infection, there was a large accumulation of cortical actin in the cell cortex and a significant increase in short, randomized filapodia-like protrusions (Figure 3J,K).

### Reduced ECM deposition and cell-cell adhesion in NKO mutant embryos

**Figure 4:**
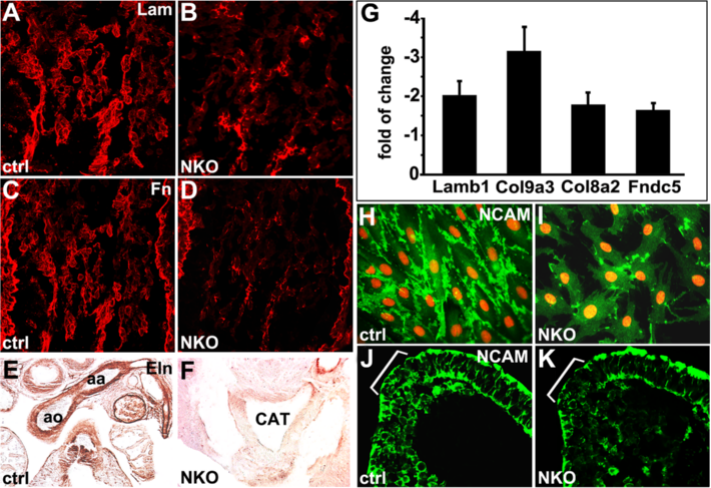
**Reduced extracellular matrix (ECM) deposition and neural cell adhesion molecule (NCAM) expression in neural crest cell (NCC)-specific integrin-linked kinase (ILK) knockout (NKO) mutants. (A-F)** Significantly reduced expression of laminin and fibronectin at E9.5 and elastin at E13 in NKO mutant OFT. **(G)** Reduced expression of Lamb1, Col9a3, Col8a2 and Fndc in NKO mutant NCCs revealed by quantitative polymerase chain reaction (qPCR) experiments. **(H-K)** NCAM expression is significantly reduced in ILK mutant NCCs, but it remains at cell-cell contacts compared to control NCCs. ILK^f/f^;Cre^+^ mutant NCCs display excessive filapodia-like protrusions **(I)**. **(J,K)** Reduced NCAM expression in dorsal neural tube of NKO mutants (brackets).

We examined whether impaired integrin-ILK signaling resulted in changes in ECM expression in NKO mutant OFTs by immunostaining. We observed significantly reduced expression of laminin and fibronectin in NKO mutant OFT at E9.5 relative to control (Figure 4A-D). At E13, elastin, a key component of the elastic matrix of the great vessels, was expressed in the aortic arch of control embryos. However, elastin expression in NKO mutant OFT was significantly reduced (Figure 4E,F). In addition, microarray analysis of RNAs from NKO mutant and control NCCs revealed significantly reduced expression of several ECM components (Additional file 2: Table S1), selected targets were confirmed by quantitative polymerase chain reaction (qPCR) studies, including lamb1, col9a3, col8a2 and Fndc5 (Figure 4G).

We further investigated whether deletion of ILK resulted in defective cell-cell adhesions by examining the expression of NCAM that is required for NCC migration [34]. In culture, control NCCs were elongated and established proper cell-cell contacts and alignment. Clusters of NCAM were found at the cell-cell contacts of control NCCs (Figure 4H). However, ILK mutant (ILK^f/f^;Cre^+^) NCCs in culture tended to be separated with markedly reduced NCAM expression (Figure 4I). Consistent with this, we observed a significant reduction in NCAM expression in the dorsal neural tube of NKO mutant embryos at E8.5 (Figure 4J,K, brackets).

### Reduced TGFβ signaling and smooth muscle differentiation in NKO mutant OFT

**Figure 5:**
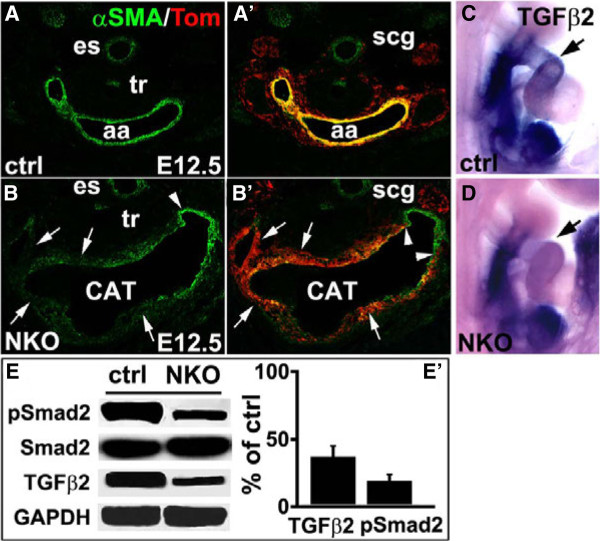
**Reduced smooth muscle differentiation and transforming growth factor β2 (TGFβ2) signaling in neural crest cell (NCC)-specific integrin-linked kinase (ILK) knockout (NKO) mutant outflow tract (OFT). (A-B’)** Reduced expression of α-smooth muscle actin (αSMA) in NKO mutant aortic arch (aa) that is derived from NCCs (tdTomato+) (B,B’, arrow) at E12.5. Smooth muscle differentiation from non-neural crest (tdTomato-) appears to be normal (B,B’, arrowhead) (*es* esophagus, *scg* superior cervical ganglia, *tr* trachea). **(C-D)** Reduced TGFβ2 expression in E9.5 NKO mutant OFT (arrow) revealed by wholemount *in situ* hybridization. **(E)** Western blot using ILK^f/f^;Cre^+^ mutant and control NCCs with antibodies to TGFβ2 and total Smad2 and phosphorylated Smad2. **(E')** quantitative assessment of western blot results (E) showing reduced TGFβ2 expression and Smad2 phosphorylation.

Differentiation of cardiac NCCs to OFT smooth muscle was assessed by immunostaining with an antibody to α-smooth muscle actin (α-SMA) and NCC lineage marker (tdTomato Red). In contrast to control OFT with well-formed smooth muscle layers (Figure 5A,A’), α-SMA expression in E12.5 NKO mutant OFT that were derived from NCCs (tdTomato Red+) was greatly reduced (Figure 5B,B’, arrow), although smooth muscle differentiation from non-NCCs (tdTomato Red-) appeared to be relatively normal (Figure 5B,B’, arrowhead). It has been reported that TGFβ2 signaling can affect OFT development [35,36], we examined TGFβ2 expression and its downstream mediator Smad2 activation at E9.5 in ILK mutants. Wholemount ISH revealed markedly reduced TGFβ2 expression in NKO mutant OFT relative to control (Figure 5C,D, arrow). In addition, western blot using ILK^f/f^;Cre^+^ mutant and control NCCs revealed significantly reduced TGFβ2 expression and Smad2 phosphorylation, although the total Smad2 level was not changed (Figure 5E, E’).

### Microarray analysis of the OFT of NKO mutants and control littermates

To gain insight into genetic programs required for NCC and OFT development, we performed microarray analysis of cardiac NCCs that were fluorescence-activated cell sorting (FACS)-sorted from the OFT of NKO mutant and control littermate embryos at E10.5 (Additional file 2: Table S1). Microarray data were submitted to the Gene Expression Omnibus database under GEO accession numbers GSE41179. Consistent with the observed phenotypes, our data revealed significant changes (fold of change ≥ ±1.5) in the expression of genes associated with migration/cytoskeleton/ECM, muscle/heart development, neurogenesis and osteogenesis (Additional file 2: Table S1).

**Figure 6:**
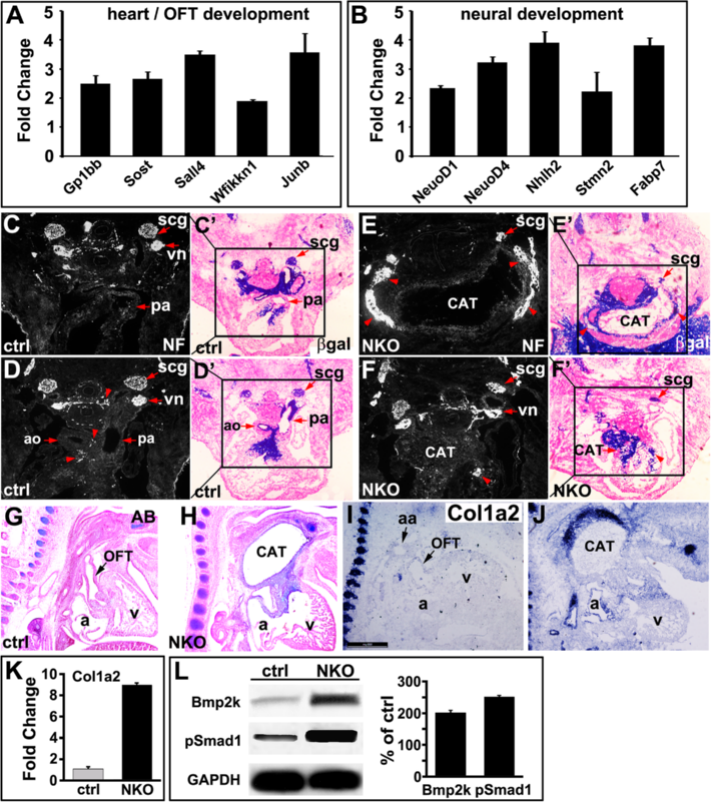
**Ectopic ossification and neurogenesis in neural crest cell (NCC)-specific integrin-linked kinase (ILK) knockout (NKO) mutant outflow tract (OFT). (A,B)** Quantitative polymerase chain reaction (qPCR) verification of upregulated genes in NKO mutant OFT. **(C-F’)** β-Galactosidase (β-gal) and neurofilament staining of adjacent sections of control (C-D’) and NKO mutant samples; (E-F’) show reduced size of superior cervical ganglia (scg) and ectopic innervation/ganglia in NKO mutant OFT ((E,F), red arrowhead). Scale bar: 200 μm; *ao* aorta, *cat* common arterial trunk, *pa* pulmonary artery, *vn* vagal nerve. **(G-J)** Ectopic ossification in the CAT and OFT cushion of NKO mutant hearts at E12.5 revealed by Alcian blue (AB) staining (G,H) and *in situ* hybridization with a probe to Col1a2 (I,J). **(K)** Increased expression of Col1a2 in NKO mutant NCCs revealed by qPCR. **(L)** Increased bone morphogenetic protein 2K (BMP2K) expression and Smad1 phosphorylation in NKO mutant NCC relative to the controls revealed by western blot.

Among genes implicated in migration/cytoskeleton/ECM, we found increased expression of Plek, Fermt3 and Ly86, but decreased expression of Col9a3, Lamb1-1, St3gal4 and Limk1. We found a significant reduction in the expression of genes of muscle differentiation including Myog, Scx, Zfp874, Xist, Neb and Nppa, suggesting reduced myocardialization of NKO mutant OFT. Sall4, a zinc finger transcription factor associated with heart and OFT development, was upregulated in NKO mutant [37]. Gp1bb, a gene located within the DiGeorge syndrome critical region, was upregulated in NKO mutants. Components of a number of signaling pathways involved in cardiovascular development were upregulated, among which, Sost and Wfikkn1 have been reported to be mediators of TGFβ, BMP and Wnt signaling, and Fabp7 is a direct target of Notch signaling in glial cells. Lbxcor1, downregulated in NKO mutants, is a Smad binding protein and negatively regulates BMP signaling. Selected targets were verified by qPCR (Additional file 2: Table S1 and Figure 6A,B).

Development of the parathyroid from the third pharyngeal pouch is initiated via inductive signals that involve NCCs. Parathyroid hypoplasia is a primary feature of DiGeorge syndrome. We found parathyroid hormone (Pth) expression was greatly reduced in NKO mutants (Additional file 2: Table S1).

### Ectopic ossification and neurogenesis in NKO mutant OFT

NCCs have the potential to differentiate into multiple cell types, including osteoblasts and neurons of the peripheral nervous system. Interestingly, in NKO mutant OFT, we found significantly increased expression of genes associated with neural differentiation, including a number of neuronal cytoskeleton and associated proteins (Sncg, Stmn2, 3, Nefm, Nefl, Tubb1) and neural signaling factors (Neurod1, Neurod4, Nhlh2, Fabp7, Elavl4) (Additional file 2: Table S1 and Figure 6B). Neurofilament immunostaining (Figure 6C,D) and β-gal staining (Figure 6C’,D’) of adjacent sections of control embryos at E12.5 revealed the formation of the superior cervical ganglia (scg) and cardiac ganglia (red arrowhead in D) around aorta and pulmonary arteries. However, in NKO mutant embryos at E12.5, we observed ectopic innervation or ganglia in the distal wall of the CAT and within the proximal wall of the OFT (Figure 6E-F’ , red arrowhead).

In addition, a number of genes associated with osteogenesis exhibited altered expression patterns, for example, expression of Spp1 was upregulated, whereas Aspn and Lect1, negative regulators of osteoblast differentiation, were downregulated, suggesting increased ossification (Additional file 2: Table S1). Consistent with this, we found, by Alcian blue (AB) staining, ectopic ossification in NKO mutant OFT at E12.5 (Figure 6G,H). In addition, ISH and qPCR revealed marked upregulation of Col1a2, a marker of osteoblast differentiation [38], in NKO mutant OFT at E12.5 (Figure 6I-K). BMP2 signaling has been implicated in osteoblast differentiation [39]. We observed significantly increased phosphorylation of Smad1 and expression of BMP2K (BMP2-inducible kinase) in the OFT of NKO mutant embryos at E10.5 (Figure 6L).

## Discussion

Our study has revealed essential roles for ILK in NCC migration and SMC differentiation within the OFT. NKO mutant embryos exhibited a distinctive enlarged common trunk (CAT) which is similar to that observed in NCC specific PINCH1 mutants. Reduced adhesion of ILK mutant NCC, reduced expression of NCAM and ECM genes, and altered TGFβ2 and BMP signaling in NKO mutant OFT may collectively contribute to the unique NCC and OFT phenotypes of NKO mutants. Microarray analysis of FACS sorted NCCs revealed decreased expression of genes associated with muscle differentiation, but increased expression of genes associated with osteogenesis and neurogenesis, suggesting a central role of ILK in integration of complex genetic programs required for NCC and OFT morphogenesis.

NCC delamination and migration to their respective final targets are timely and coordinated processes regulated by cell-cell, cell-ECM interactions and cytoskeleton organization. Ablation of ILK resulted in precocious post otic NCC emigration at E8.5 (5 to 6 somites), when few, if any, control postotic NCCs had started to emigrate. At E9.0, cranial NCCs (including cardiac NCCs) of control embryos migrated as distinct streams to the pharyngeal arches and circumpharyngeal region. However, a markedly increased number of NCCs were found in the pharyngeal arches and the aortic sac, some of which had prematurely migrated deep into the truncal OFT. At E10.5, a marked increase in NCC numbers was found in mutant pharyngeal arches. Within the OFT, control NCCs at E10.5 migrated as two distinct columns within the subendocardial cushion up to the conotruncal junction [40]. In contrast, NCCs in the OFT of NKO mutants were significantly increased, and migrated deeper into the conal region. However, no changes in neural crest cell proliferation and cell death were observed in NKO mutant OFTs compared to control littermates. These observations suggest that deletion of ILK leads to increased NCC motility and the loss of a distinct distribution pattern of NCCs within the OFT. Consistent with this, in neural tube explant cultures, outgrowth of control NCCs started 24 h after incubation as distinct streams. However, migration of NKO mutant NCCs started earlier and they lost their stream-like pattern. NKO mutant NCCs in culture migrated faster and to a bigger outgrowth area, and tended to migrate as individual cells with less cell-cell contacts and directionality. Furthermore, control NCCs were polarized with well-aligned actin filaments, whereas NKO mutant NCCs exhibited markedly disorganized actin filaments with increased cortical actin protrusions, suggestive of increased motility [41]. In addition, NKO mutant NCCs in culture exhibited a significant reduction in integrin activation, focal adhesion complex formation and Akt activation. Furthermore, in dorsal neural tube and cultured NCCs, we observed markedly reduced expression of NCAM required for the emigration and early migration of NCCs [34]. These observations revealed a critical requirement for ILK in modulating the timing, velocity and pattern of NCC migration.

Numerous molecules, including growth factors, ECM and adhesion molecules play essential roles in pharyngeal arch artery and OFT morphogenesis. Loss of individual genes commonly causes certain parts of pharyngeal arteries to abnormally persist or regress, leading to a spectrum of OFT anomalies. However, most mutant mice exhibit no defects in cardiac NCC delamination and migration. For example, NCCs deficient in BMP, PDGF and TGFβ signaling, fibronectin, α5-integrin, PINCH1, FAK and N-cadherin populate the pharyngeal arches and OFT properly, although subsequent NCC proliferation, survival and differentiation within the OFT are variably impaired, affecting subsets of NCCs [8-10,13,14,36,42,43]. However, in NKO mutants, we observed marked changes in the timing and pattern of NCC migration without significant changes in proliferation and cell death, suggesting a distinct role for ILK in NCC and OFT morphogenesis.

NCCs have the potential to differentiate into multiple cell types, including cartilage, vascular SMC and peripheral neurons, which involves multiple signaling pathways, including TGFβ2 and BMP signaling [1]. NKO mutant mice exhibited an enlarged common arterial trunk that appeared to be formed by fusion of aortic arches and proximal parts of pharyngeal arteries. Impaired SMC differentiation was observed that affected all segments of NKO mutant OFT. Therefore, in contrast to previous studies in which defective SMC differentiation affects only subsets of NCCs and their contributing pharyngeal arch arteries or aortic arches [4,8,14,44], the failure of SMC differentiation in NKO mutant OFT is pervasive. Reduced TGFβ2 signaling but increased BMP2 signaling was observed in NKO mutant OFTs that may account in part for the observed OFT phenotype. Loss of TGFβ2 signaling has been shown to lead to ectopic great vessel innervation and ganglion formation within the OFT [36], whereas BMP2 signaling is required for osteoblastic differentiation [39]. Consistent with this, we observed ectopic ossification and neurogenesis in NKO mutant OFT. Furthermore, our microarray analysis of FACS purified NCCs revealed significantly increased expression of genes involved in neurogenesis (Neurod1, Neurod4, Nefm, Nefl, Tubb1, Scn9a) and osteogenesis (Spp1, Sost), suggesting a role for ILK in cardiac NCC differentiation and fate determination.

A number of genes associated with muscle differentiation (Myog, Scx, Zfp874, Xist, Neb and Nppa) were downregulated in NKO mutant OFT, suggesting impaired myocardialization of NKO mutant OFT. Sall4 is a zinc finger transcription factor upregulated in NKO mutants, and mutation of Sall4 causes ventricular septal defects [37]. DiGeorge syndrome is a common congenital disorder caused by chromosome 22q11 deletion resulting in defective neural crest migration. A region containing four genes (Wdvcf, Tbx1, Gp1bb and Pnutl) has been identified responsible for cardiac outflow tract defects and lethality. Although Tbx1 is the major candidate gene for DiGeorge syndrome, other genes are likely to function as modifiers [45,46]. Gp1bb is a gene located within the DiGeorge syndrome critical region. Deletion or reduced expression of Gp1bb is associated with a human bleeding disorder, Bernard-Soulier syndrome [46,47].

## Conclusions

Our study has revealed an essential role for ILK in integrating signaling pathways and the crosstalk between ECM and cytoskeleton required for NCC and OFT morphogenesis. Defects in these interactions results in abnormal NCC migration and differentiation that contribute to the unique NKO mutant OFT phenotype. Future studies are needed to further elucidate how ILK and PINCH1 may cooperate or interact with each other to regulate neural crest migration and differentiation, whether these defects are related or two separate processes, and how each process contributes to observed phenotypes. It also remains to be determined whether ILK switches NCCs from a cardiac fate to a cranial fate to form cranial-specific cartilage cells.

## Methods

### Mice

To generate neural crest conditional ILK knockout (NKO) mice, mice homozygous for the ILK floxed allele (ILK^f/f^) [27] were mated with Wnt1-Cre mice to generate mice with Wnt1-Cre and heterozygous ILK floxed allele (ILK^f/+^). NKO (Wnt1-Cre; ILK^f/f^) mice were generated by backcrossing these mice to ILK^f/f^ mice. For *in vivo* fate mapping study of NCCs, Wnt1-Cre; ILK^f/+^ mice were mated with ILK^f/f^ mice carrying a Rosa-LacZ or tdTomato Red reporter mice [31,32]. All experiments involving mice were carried out according to a protocol reviewed and approved by the Institutional Animal Care and Use Committee of USCD, USA and approved by the Animal Committee of Tongji University School of Medicine, China.

### Histology and *in situ* hybridization

Females with copulation plugs were considered to be at embryonic development day 0.5 (E0.5) of gestation. Pregnant females were killed at different stages of gestation, and embryos were dissected for morphologic and histological analysis as described [13]. For β-gal staining, embryos expressing β-gal were harvested in cold phosphate-buffered saline (PBS) and fixed for 1 to 2 h in 4% paraformaldehyde (PFA) and incubated in β-gal substrate as described [13]. For high-resolution analysis of β-gal activity, embryos were paraffin embedded, sectioned, and counterstained with nuclear Fast Red.

Whole amount *in situ* hybridization was carried out with digoxigenin-labeled RNA probes as previously described [13,24]. 5-Bromodeoxyuridine (BrdU) labeling and apoptosis assays were performed as described [24].

For Alcian Blue staining, deparaffinized and hydrated sections were stained in AB solution for 30 minutes, followed by washing in running tap water for 2 minutes, and counterstained in nuclear fast red solution for 5 minutes.

### Immunostaining and western blot

Immunostaining was performed as described [13,48]. 10 μm sections and isolated cells were incubated with primary antibodies overnight at 4°C. The following primary antibodies were used: α-SMA (Abcam, ab7817), phosphohistone H3 (Cell Signaling, #9701), BrdU (Abcam), β-gal (Cappel, #55976), ILK (Sigma, I0783), laminin (Abcam, ab11575), fibronectin (Abcam, ab2413), elastin (Sigma, E4013), NCAM (Millipore, AB5032), TGFβ2 (Abcam, ab15539), p-Akt (Cell signaling, #9271), p-Smad1 (Cell Signaling, #9516), p-Smad2 (Abcam, ab5478), Sox10 (Abcam, ab27655), BMP2K (Abcam, ab115469), neurofilament (Developmental Studies Hybridoma Bank, 2H3), glyceraldehyde 3-phosphate dehydrogenase (GAPDH) (Santa Cruz Biotechnology), parvin (Abcam) and paxillin (Abcam). After washing with 0.25% TritonX-100 in PBS, sections were incubated with either fluorescently labeled (Molecular Probes, Invitrogen) or biotinylated (Vector) secondary antibodies for 2 h.

TUNEL staining was performed as recommended (Roche). For BrdU staining, timed-pregnant mice at were injected with 500 μl of BrdU (proprietary mixture, Ambion) and BrdU staining was performed as described [49]. For cell counting, OFT sections of the appropriated developmental stages were cut at 10 μm, and every fourth section was stained and cells doubly positive for tdTomato Red and Ph3 (or BrdU^+^ or TUNEL^+^) were counted. Mitotic indices were determined by counting the number of cells per section that were doubly positive for tdTomato Red (NCCs) and Ph3 staining, normalized to the total number of NCCs and derivatives (tdTomato+) within pharyngeal arteries and OFTs of NKO mutant and control embryos.

Western blotting was performed as described [48]. Expression levels of proteins and RNA were quantified in Photoshop.

### Neural crest cultures and adhesion assay

We performed neural crest cell cultures as described [50]. Briefly, embryos were collected at E8.5 (4 to 10 somites) and treated with 0.5 mg/ml of collagenase/dispase and then bisected longitudinally to expose the hindbrain neural folds. The portion of neural tube between the otic placode and the third somite was transected and cultured in a 35-mm fibronectin-coated Petri dish containing high glucose DME and 10% FBS. Cultures were maintained for 72 h at 37°C with 5% CO_2_, and then each neural tube explant was removed. Neural crest cell outgrowths attached to the culture were used for analysis. For NCC outgrowth analysis, neural tube explants (n = 3) were embedded in metrigel (BD Biosciences) on a coverslip and cultured for 24 h. Explant cultures were fixed in 4% PFA and confocal images of the cultures were taken. NCC outgrowth area (migration index) and migration distances were measured and analyzed using Leica image software as described [33]. Three areas per explants cultures were analyzed.

To establish ILK knockout NCC culture, the cultured ILK^f/f^ NCCs were infected with 10^7^ pfu/well of either control β-gal or Cre adenovirus overnight. The medium was changed and cells were cultured for additional 48 h before cell adhesion assay. For cell adhesion assays, ILK^f/f^ or control NCCs were serum starved overnight and detached with trypsin and resuspended in assay buffer (137 mM NaCl, 2.7 mM KCl, 1 mM MgCl_2_•6H_2_O, 3.3 mM NaH_2_PO_4_•H_2_O, 20 mM HEPES (*N*-2-hydroxyethylpoperazine-*N*-2-ethanesulfonic acid), pH 7.4, 0.1% bovine serum albumin). Microtiter plates (Nunc) were coated with matrix proteins (5 μg/ml) overnight at 4°C, washed with PBS and blocked with 1% heat-denatured bovine serum albumin (BSA) for 1 h at room temperature. Cells were counted with a hemocytometer and 20,000 cells per well were added to matrix-coated microtiter plates and incubated for 15 minutes at 37°C. Cells were washed three times with assay buffer and the number of attached cells determined by acid phosphatase assay [51].

### MRI

Embryos were analyzed by MRI as previously described [13]. Briefly, paraformaldehyde-fixed embryos were embedded in a MRI contrast agent and imaged using an 11.7 Tesla magnet. The 3D MRI dataset obtained was reconstructed into axial TIFF slices. Image datasets were analyzed and 3D reconstructions created using Amira 3.0 software.

### Microarray and qPCR

The OFTs of NKO mutant and control embryos at E10.5 were dissected and the samples for the same genotype were pooled, digested with a mixture of collagenase/dispase (1 mg/ml, Roche)/trypsin (0.1%) for 10 minutes with periodical pipetting, and clear supernatants were collected in a 15 ml tube. The digestion was repeated until OFT tissue was totally digested. The cell suspension was filtered through a 40 μm filter unit (Fisher), centrifuged and resuspended in up to 0.5 ml staining medium, and stained with FDG using FluoReporter LacZ Flow Cytometry kits (Molecular Probes, F-1930) following the manufacture’s instruction. The treated cell suspensions were FACS sorted (BD FACSAria) and stored at -80°C. After multiple cell sorting until enough cells were obtained, cells from individual collections were pooled at the time of RNA extraction. RNA was prepared using the RNeasy Mini kit (Qiagen) following the manufacturer’s instructions. For microarray analysis, aRNAs were synthesized using the Message Amp II kit (Ambion) following the manufacturer’s instructions for a one-step amplification and hybridized to GeneChip Mouse Genome 430 array (Affimetrix) following the protocol. The microarray data have been submitted to NCBI GEO database under accession number GSE41179. The expression data were normalized and differential expression was defined based on a cutoff of 1.5 fold change (FC). Candidate targets of interest were verified by qPCR using SYBR green detection. Primer pairs for exon specific PCR appear in Additional file 3: Table S2.

### Statistical analysis

Data were presented as mean ± SEM and the Student t test was used for two-group comparisons. Differences were considered statistically significant at a value of *P* <0.05.

## Abbreviations

AB: Alcian blue; ao: Aorta; aoa: Aortic arch; as: Aortic sac; CAT: Common arterial trunk; da: Ductus arteriosus; ECM: Extracellular matrix; FAK: Focal adhesion kinase; ILK: Integrin-linked kinase; ISH: *in situ* hybridization; lv: Left ventricle; NCC: Neural crest cell; NKO: NCC-specific ILK knockout; OFT: Outflow tract; pa: Pulmonary artery; px: Pharynx; rv: Right ventricle; RVOT: Right ventricular outflow tract; scg: Superior cervical ganglia; SMC: Smooth muscle cell; VSD: Ventricular septal defect.

## Competing interests

The authors declare that they have no competing interests.

## Authors’ contributions

XD, WJ, QZ, YS and XL conceived, designed and performed the experiments, analyzed the data and drafted the manuscript. LX, PG, SZ, BGP, CJ, LP and SB performed the experiments and analyzed the data. SME and JC conceived and designed the study, and helped to draft the manuscript. All authors read and approved the final manuscript.

## Supplementary Material

Additional file 1: Figure S1 Expression of integrin-linked kinase (ILK) in the outflow tract (OFT) and neural crest cells (NCCs). (A, B’) Wholemount in situ hybridization show ubiquitous ILK expression in E8.5 and E9.5 embryos. (C-E) Immunostaining with ILK antibody and β-gal staining of adjacent sections from Wnt1-Cre; R26R embryos at E9.5 show that ILK expression in cardiac NCC in the OFT. (F-H) Immunostaining with antibodies to ILK and Sox10 show ILK expression in cultured NCCs. (I-L) ILK is not detected in cardiac NCC-derived OFT mesenchyme in E9.5 NKO mutants. Click here for file

Additional file 2: Table S1 Microarray analysis of neural crest cells from the outflow tract (OFT) of control and integrin-linked kinase (ILK) mutant embryos at E10.5. Microarray and quantitative polymerase chain reaction (qPCR) analysis of neural crest cells from the OFT of control and ILK mutant embryos at E10.5. Click here for file

Additional file 3: Table S2 Quantitative polymerase chain reaction (qPCR) primer list. List of qPCR primers used in this study. Click here for file
