# Supplementary figures and images for: Requirement for integrin-linked kinase in neural crest migration and differentiation and outflow tract morphogenesis
Source: BMC Biol. 2013 Oct 16;11:107. doi: 10.1186/1741-7007-11-107 (PMC3906977; doi:10.1186/1741-7007-11-107)

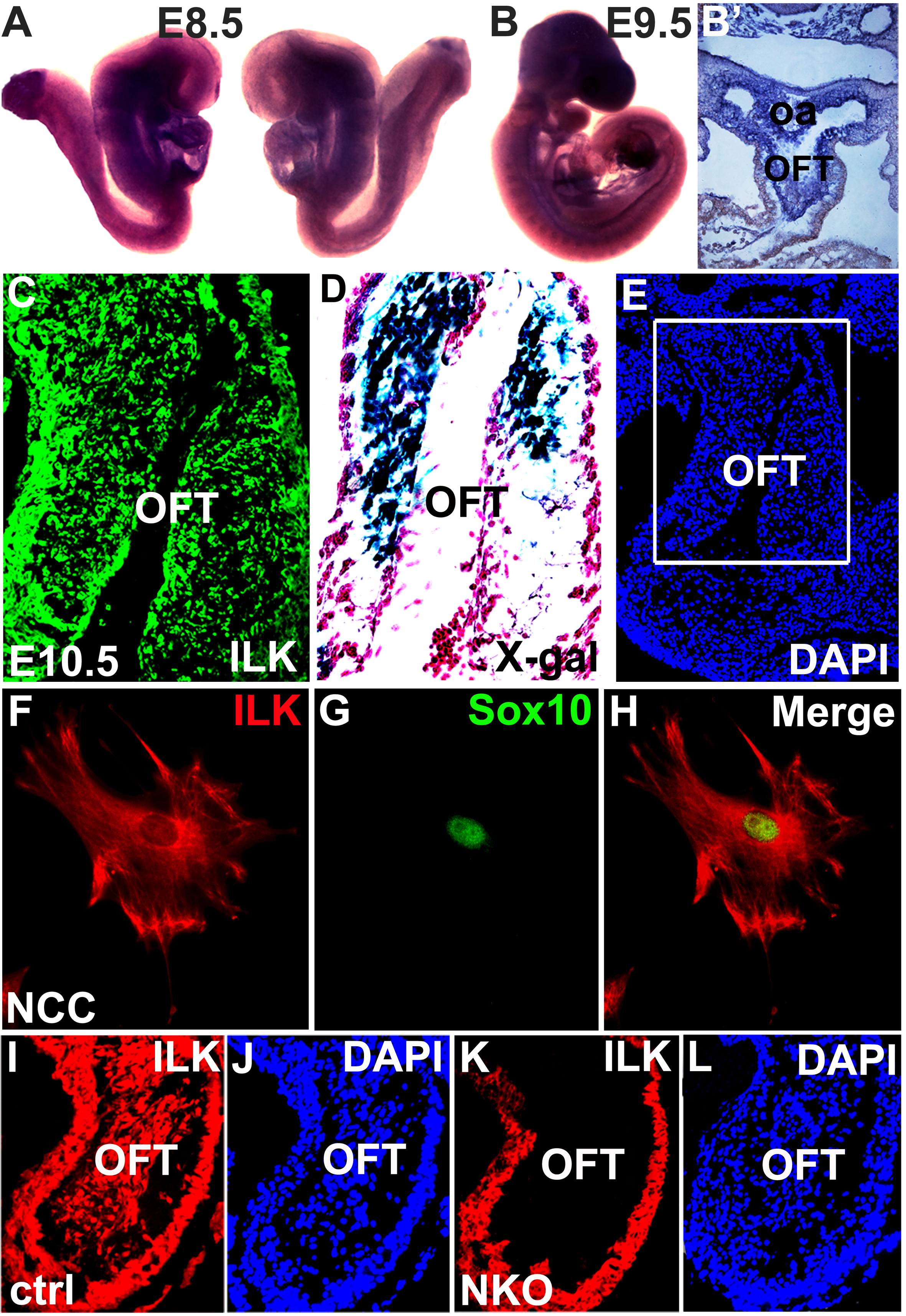

Supplement: Additional file 1: Figure S1 — Expression of integrin-linked kinase (ILK) in the outflow tract (OFT) and neural crest cells (NCCs). (A, B’) Wholemount in situ hybridization show ubiquitous ILK expression in E8.5 and E9.5 embryos. (C-E) Immunostaining with ILK antibody and β-gal staining of adjacent sections from Wnt1-Cre; R26R embryos at E9.5 show that ILK expression in cardiac NCC in the OFT. (F-H) Immunostaining with antibodies to ILK and Sox10 show ILK expression in cultured NCCs. (I-L) ILK is not detected in cardiac NCC-derived OFT mesenchyme in E9.5 NKO mutants. [file 1741-7007-11-107-S1.tif]
