# Supplementary material for: Requirement for integrin-linked kinase in neural crest migration and differentiation and outflow tract morphogenesis
Source: BMC Biol. 2013 Oct 16;11:107. doi: 10.1186/1741-7007-11-107 (PMC3906977; doi:10.1186/1741-7007-11-107)
Supplement: Additional file 2: Table S1 — Microarray analysis of neural crest cells from the outflow tract (OFT) of control and integrin-linked kinase (ILK) mutant embryos at E10.5. Microarray and quantitative polymerase chain reaction (qPCR) analysis of neural crest cells from the OFT of control and ILK mutant embryos at E10.5. [file 1741-7007-11-107-S2.pdf]

## Additional file 2

### Table S1: Microarray analysis of neural crest cells from the OFT of control and ILK mutant embryos at E10.5

| gene | description | ctrl signal | FC(mut/ctrl) | |
|---|---|---|---|---|
| | | | Microarray | qPCR |
| **muscle differentiation** | | | | |
| Myog | myogenin | 449.7 | 0.667 | 0.23 |
| Scx | scleraxis | 318.6 | 0.659 | 0.65 |
| Zfp874 | zinc finger protein 874 | 164.8 | 0.656 | |
| Xist \| Xist | inactive X specific transcripts | 1038 | 0.652 | |
| Neb | nebulin | 78.82 | 0.638 | |
| Nppa | natriuretic peptide precursor type A | 629.8 | 0.621 | |
| **heart and outflow tract development** | | | | |
| Gp1bb | glycoprotein Ib, beta polypeptide | 73.1 | 1.5 | 1.93 |
| Sall4 | sal-like 4 (Drosophila) | 454.8 | 1.487 | 3.49 |
| Sost | sclerostin | 255.5 | 1.458 | 2.67 |
| Wfikkn1 | WAP, FS, Ig, KU and NTR-containing protein 1 | 80.18 | 1.555 | 1.8 |
| Edn1 | endothelin 1 | 59.69 | 1.74 | |
| Junb | Jun-B oncogene | 56.07 | 1.45 | 3.56 |
| Zic1 | zinc finger protein of the cerebellum 1 | 103.7 | 0.548 | 0.68 |
| **neural development** | | | | |
| Scn9a | sodium channel, voltage-gated, type IX, alpha | 13.57 | 4.089 | 5.62 |
| Sncg | synuclein, gamma | 113.8 | 3.038 | |
| Stmn3 | stathmin-like 3 | 119.9 | 2.771 | |
| Stmn2 | stathmin-like 2 | 110.9 | 2.156 | 2.75 |
| Nefm | neurofilament, medium polypeptide | 401.5 | 2.024 | |
| Neurod1 | neurogenic differentiation 1 | 113.7 | 1.974 | 2.32 |
| Nefl | neurofilament, light polypeptide | 572.6 | 1.788 | |
| Tubb1 | tubulin, beta 1 | 107.2 | 1.724 | |
| Ina | internexin neuronal intermediate filament protein, alpha | 97.78 | 1.697 | |
| Neurod4 | neurogenic differentiation 4 | 63.81 | 1.667 | 2.33 |
| Elavl4 | ELAV (embryonic lethal, abnormal vision, Drosophila)-like 4 (Hu antigen D) | 147.3 | 1.664 | |
| Prtg | protogenin homolog (Gallus gallus) | 602.3 | 1.663 | |
| Fabp7 | fatty acid binding protein 7, brain | 386.5 | 2.114 | 3.65 |
| Nhlh2 | nescient helix loop helix 2 | 84.6 | 1.514 | 3.93 |
| **Osteogenesis** | | | | |
| Spp1 | secreted phosphoprotein 1 | 99.57 | 1.831 | |
| Junb | Jun-B oncogene | 56.07 | 1.45 | 3.56 |
| Aspn | asporin | 90.61 | 0.656 | |
| Sost | sclerostin | 255.5 | 1.458 | 2.67 |
| Lbxcor1 | ladybird homeobox 1 homolog (Drosophila) corepressor 1 | 98.64 | 0.628 | |
| Lect1 | leukocyte cell derived chemotaxin 1 | 90.35 | 0.625 | |
| **Migration and cytoskeleton** | | | | |
| Plek | pleckstrin | 67.86 | 1.586 | 2.5 |
| Fermt3 | fermitin family homolog 3 (Drosophila) | 235.8 | 1.579 | 1.48 |
| Ly86 | lymphocyte antigen 86 | 83.32 | 1.536 | |
| Col9a3 | collagen, type IX, alpha 3 | 105.3 | 0.634 | 0.3 |
| Limk1 | | 63.22 | 0.618 | |

| | | | | |
|---|---|---|---|---|
| Ilk | integrin linked kinase | 805.1 | 0.531 | |
| Lamb1-1 | laminin B1 subunit 1 | 52.01 | 0.518 | 0.5 |
| St3gal4 | ST3 beta-galactoside alpha-2,3-sialyltransferase 4 | 97.51 | 0.301 | |
| **others** | | | | |
| Taf10 | TAF10 RNA polymerase II, TATA box binding protein (TBP)-associated factor | 83 | 2.879 | |
| Skiv2l2 | superkiller viralicidic activity 2-like 2 | 198.3 | 1.573 | |
| Atad5 | ATPase family, AAA domain containing 5 | 77.42 | 1.571 | |
| Trim71 | | 192.7 | 1.559 | |
| Zhx1 | zinc fingers and homeoboxes 1 | 78.71 | 1.538 | |
| Cuedc1 | CUE domain containing 1 | 111.9 | 1.537 | |
| Fnbp4 | formin binding protein 4 | 82.42 | 1.504 | |
| Pth | parathyroid hormone | 155.7 | 0.391 | |
| Afp | alpha fetoprotein | 80.15 | 0.311 | |

Note: The expression data were normalized and differential expression was defined based on a cutoff of 1.5 fold change (FC) with a *p* value ≤ 0.05.
