# Supplementary material for: Requirement for integrin-linked kinase in neural crest migration and differentiation and outflow tract morphogenesis
Source: BMC Biol. 2013 Oct 16;11:107. doi: 10.1186/1741-7007-11-107 (PMC3906977; doi:10.1186/1741-7007-11-107)
Supplement: Additional file 3: Table S2 — Quantitative polymerase chain reaction (qPCR) primer list. List of qPCR primers used in this study. [file 1741-7007-11-107-S3.pdf]

Additional File 3

Table S2: qPCR primer List

Oligo Name	Oligo Sequence (5' -- 3')	Oligo Name	Oligo Sequence (5' -- 3')
Scn9a		Gp1bb	
forward	CAACGCACTCATAGGAGCAA	Forward	ACCCTATCTGCCTTCGCAAC
reverse	CTTGCCAGCAAACAGATTGA	Reverse	TAGAAAGCTGCAGACCCGAC
Scx		Sost	
forward	CCCAAACAGATCTGCACCTT	Forward	AGCCTTCAGGAATGATGCCAC
reverse	TGTCACGGTCTTTGCTCAAC	Reverse	CTTTGGCGTCATAGGGATGGT
Col9a3		Wfikkn1	primer pair 1
forward	GGAGAACGGGGAAGTCTAGG	Forward	TTTGATCCCTGTTGGGCTCC
reverse	CACGTAGACCAATCCCACCT	Reverse	TGGTCAGCATAGGACTGGGA
			primer pair 2
Lamb1-1			AACAGCAATAACTTTGAGACCCG
forward	GTTCGAGGGAACTGCTTCTG		TCGTCCTTTAGCACATCATCCA
reverse	GTTCAGGCCTTTGGTGTTGT		
		Sall4	
Bmp2k		Forward	TTGCGACCACCCAAGTAT
forward	CCTACCAGCGCAAAGAAGAC	Reverse	AACCCACAGAACCAACCAC
reverse	TCTCCTTGGAATCGGAAATG		
		Junb	
Col8a2		Forward	CTACAAACTCCTGAAACCCACC
forward	AGGGTCCAGTAGGGGCTAAA	Reverse	TCTGATCCCTGACCCGAAA
reverse	CCCTTAGGTCCTGGTTTTCC		
		Junb	
Myog		Forward	CTACAAACTCCTGAAACCCACC
forward	ACCAGGAGCCCCACTTCTAT	Reverse	TCTGATCCCTGACCCGAAA
reverse	GTCCCCAGTCCCTTTTCTTC		
		Zic1	
Plek		Forward	GCAAGATGTGCGATAAGTCC
forward	CTTGAGAGGCTGTGTGGTGA	Reverse	CGGGTTGTCTGTTGTGGG
reverse	CTGGATGGCTTTGATCCACT		
		Fermt3	
Fndc5		Forward	ATGGCGGGTATGAAGACAGC
forward	GGTGCTGATCATTGTTGTGG	Reverse	CACCAATGTGCGACTCCCC
reverse	GGCTCGTTGTCCTTGATGAT		
		Skiv2l2	

Stmn2		Forward	AAGTTATGCGAGAAGTTGCTTGG
forward	GCCGCGCAACTACAACATC	Reverse	AGCATTTGGAATAGTAGCCGAAA
reverse	CCCCCTGGAGAAAGTTACCTT	Neurod1	
		Forward	TTCACGATTAGAGGCACG
Fabp7		Reverse	TCCAAAGGCAGTAACGAC
forward	GGACACAATGCACATTCAAGAAC		
reverse	CCGAACCACAGACTTACAGTTT	Neurod4	
		Forward	TGCCTTAGCACTTCTTTCT
Nhlh2		Reverse	GTGTTATGGGTCTGGTTTC
Forward	CAACCATACACCCGCTAA		
Reverse	CCAGGAAACAAGGAGGAG		
